# Case report: a common trunk of the coronary arteries

**DOI:** 10.1007/s00276-016-1736-4

**Published:** 2016-08-25

**Authors:** Erich Brenner, Elisabeth Pechriggl, Marit Zwierzina, Romed Hörmann, Bernhard Moriggl

**Affiliations:** Division for Clinical and Functional Anatomy, Medical University of Innsbruck, Müllerstrasse 59, 6020 Innsbruck, Austria

**Keywords:** Coronary arteries, Anatomical variation, Mixed trunk

## Abstract

We describe the heart from a 79-year-old woman with no medical history of cardiac complaints. Her heart shows a regular right coronary artery (RCA) and a variant left coronary artery (LCA) arising from the right sinus of Valsalva. The common stem of the RCA and the LCA is extremely short. The LCA depicts a preinfundibular course with a cranial-anterior loop and reaches the intersection of the anterior interventricular sulcus and the left coronary sulcus, where it divides into the regular branches, the anterior interventricular branch (left anterior descending, LAD) and the circumflex branch (left circumflex, LCx). All further branching resembles a normal distribution with the posterior interventricular branch coming for the RCA. Such a variant LCA is extremely rare with a reported incidence of 0.17 %. However, recognition and angiographic demonstration of such a variation assume the highest priority in a patient undergoing, for instance, direct coronary artery surgery or prosthetic valve replacement.

## Introduction

Among the variations of the coronary arteries, the presence of a common trunk of the coronary arteries is well known [1, 11, 27]. The hitherto largest study on this topic found 1.3 % of congenital coronary artery anomalies in 126,595 patients undergoing coronary arteriography [1, 30]. Nevertheless, angiological studies reported more coronary anomalies than anatomical studies, as angiographies are performed only on patients with clinical signs [11]. Therefore, such studies are biased by selection.

Variations and anomalies of the coronary arteries are of clinical interest as unrecognized coronary variations and anomalies may lead to errors in diagnoses, and surgical problems may follow if a variant or anomalous coronary artery is excluded from perfusion during open heart surgery, or if it is unwittingly incised by the surgeon [27].

## Method

After excision from the corpse, the isolated heart was dissected in detail to isolate the coronary vessels completely. This dissection allowed for a continuous documentation.

Prior to dissection, the two coronary arteries were injected from their common origin with a mixture of a red, hardening latex and a barium sulfate solution as contrast agent. To prevent the solution from leaking out, gaps in the vessels’ walls were closed with wax. The injection solution facilitates the appropriate dissection and CT imaging of vessels.

The documentation was carried out with photographs, film sequences, and CT scans (Discovery CT750 HD, GE Medical Systems). Measurements were taken from multi-planar reconstructions of these scans. Furthermore, three-dimensional reconstructions of the vessels were calculated (Slicer Dicer©, Ver 5.2, Pixotec, LLC, Renton, WA, USA).

## The case

This case of a common trunk of the coronary arteries was found during the regular topographical dissection course for second-year students in the winter term 2014/15 in a 79-year-old woman [ID 2/056], who died in 2012 from ovarian and lung cancer. No cardiac complaints were known.

The body was donated to the Division of Clinical and Functional Anatomy of the Medical University of Innsbruck [14, 23].

The cadaver was preserved using an arterial injection of a formaldehyde-phenol solution and consecutive immersion in phenolic acid in water for about 3 months [20]. A recent analysis showed that bodies donated to our division are a representative sample of the general Austrian population at the age of death [10].

**Fig. 1:**
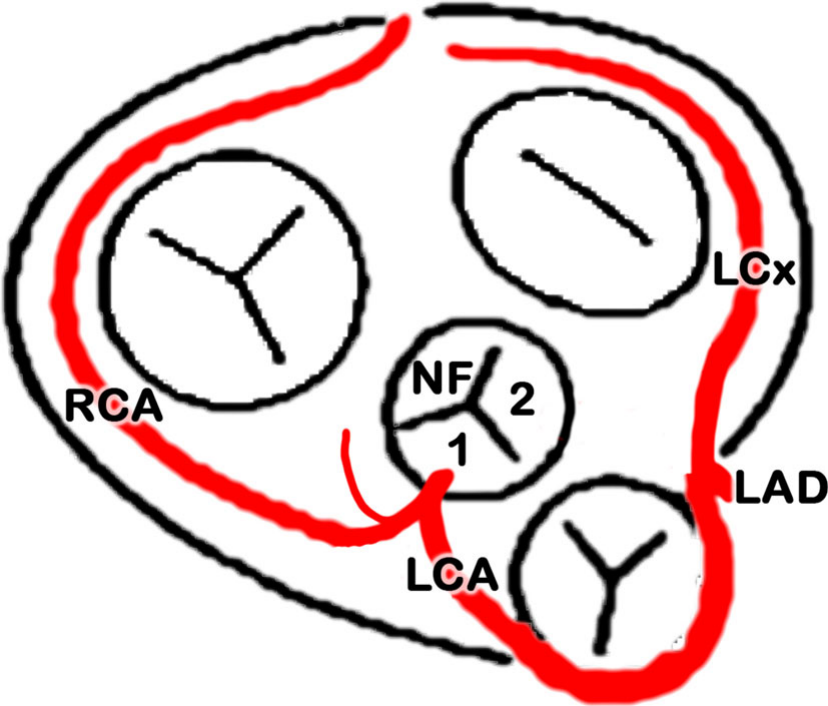
Current case, schematic drawing according to the style by Lippert and Pabst, 1985 [11]; the left coronary artery (LCA) arises from a common trunk together with the right coronary artery (RCA) and passes ventrally to the conus arteriosus (preinfundibularly). *LAD* left anterior descending, *LCx* left circumflex; *1* sinus #1 (*right*); *2* sinus #2 (*left*); *NF* non-facing sinus

**Fig. 2:**
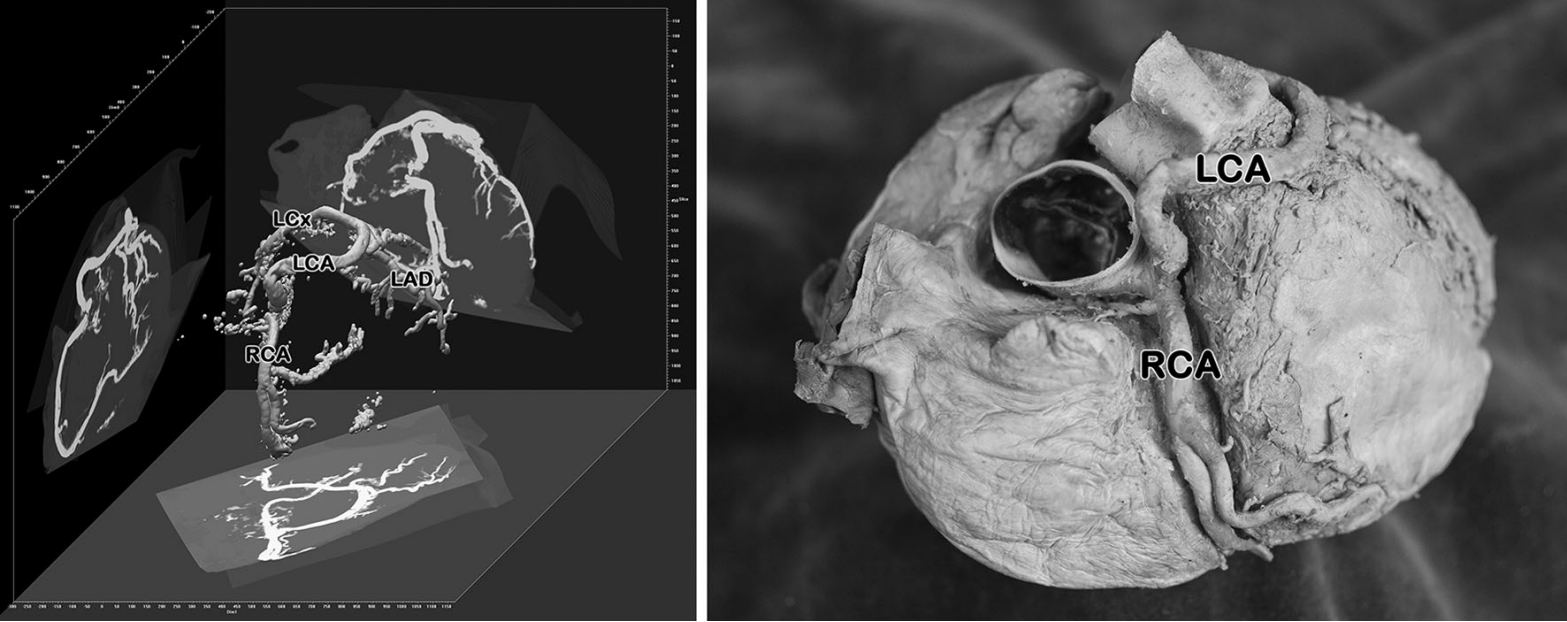
Current case, 3D-reconstruction of serial CT scans (*left*) and corresponding view of the isolated specimen (*right*). *RCA* right coronary artery, *LCA* left coronary artery, *LAD* left anterior descending, *LCx* left circumflex

The right sinus of Valsalva gives rise to an extremely short [outer surface: 8 mm; luminal: 4 mm] common (or mixed) trunk [single coronary type II from sinus #1; 17], which immediately divides into a regular right coronary artery (RCA) and a variant left coronary artery (LCA) (Figs. 1, 2). The ostium is slightly oval (6 × 7 mm). The RCA shows a straight course for about 20 mm with a diameter of about 4 mm and then turns into the right coronary sulcus at an angle of about 55°; the sinoatrial branch leaves at a distance of about 6 mm. The LCA turns after about 10 mm (diameter 5 mm) approximately 70° upwards along the ascending aorta forming a cranial-anterior loop with a 110° bend, then runs gently curved anteriorly around the pulmonary trunk and reaches the intersection of the anterior interventricular sulcus and the left coronary sulcus, where it divides into the regular branches, the anterior interventricular branch (left anterior descending, LAD) and the circumflex branch (left circumflex, LCx). All further branching resembles a normal distribution with the posterior interventricular branch coming for the RCA. According to the Leiden convention, this anomaly could be described as 1R LCx;2- [7].

Detailed analysis of the serial CT scans revealed no further—intracardiac—anomaly of this heart.

## Discussion

According to Muresian [16], “*the transition from ‘variation’ toward ‘anomaly’ is gradual and precise limits are impossible to be set. Anomalies should, however, be seen in the context of the heart and of the cardiovascular system: probably pure or singular anomalies do not exist (when single, the term ‘variation’ probably would be more suited).*” As this case resembled a real singular situation, we would like to term this a ‘variant LCA’, whereas in the literature the terms “anomaly” and “variation” are mainly mixed with “anomaly” in favor.

### Descriptions in the literature

There are several descriptions of an anomalous origin of the LCA from the right sinus of Valsalva, either as a separate vessel or in common with the RCA. In most of these cases, the LCA or its branches ran either posteriorly to the aorta or between the aorta and the pulmonary trunk. When there is a vessel passing preinfundibularly, it is mostly the LAD [5].

Smith was the first to classify single coronary arteries [26]. Type 1 summoned single coronary arteries that followed the distribution pattern of only one coronary artery [Type I; 17]. Type 2 summoned single coronary arteries with an almost immediate division into right and left branches and almost normal distribution of both coronary arteries [Type II; 17]. Type 3 comprised those cases that had an atypical distribution of the coronary arteries [Type III; 17].

Ogden and Goodyer [18] proposed another classification composed of a letter, either “R” for “origin from the right sinus” or “L” for “origin from the left sinus”, followed by a number (1–4 for L, 1–5 for R), and—in the case of R by a minor letter (a–c). According to this classification, our case would be of Type R-4a; “*In type 4a there is a normal distal right coronary artery, while the main left coronary artery arises as one of the initial branches of the right artery, crosses the ventricular outflow tract anterior to the great vessels, and subsequently divides into circumflex and anterior descending branches […].*” They found this variation in 12 cases out of altogether 142 cases of a single coronary artery; as a pity there is no relation to the general population and, therefore, incidence cannot be appreciated.

Engel and co-workers found one LCA (main left coronary artery) out of 4250 angiographies arising from the right sinus of Valsalva (0.024 %) and running anteriorly to the aorta, but obviously not anteriorly to the pulmonary trunk [6].

In their extensive analysis of 126,595 angiographies, Yamanaka and Hobbs found an LCA (left main trunk) arising from the right sinus of Valsalva in 22 cases (0.017 %) [30]. These authors distinguished five anatomical subtypes according to the relationship of the anomalous coronary artery with the aorta and pulmonary artery, i.e., “anterior,” “between,” “septal,” “posterior,” and “combined.” In their series, the “septal” subtype was the most common, whereas the “between” type was rare. As a matter of fact, the “anterior” subtype, similar to our case, was described, but not quantified: “*In the third anatomical subtype, the left main [coronary artery] passes anterior to the pulmonary artery […]. The anomalous vessel follows a pattern similar to the conus branch with a cranial-anterior loop […]. The LMT then divides at the junction of the proximal and middle thirds of the septum. […] This anomaly usually is benign, although instances of angina pectoris and myocardial infarction in the absence of coronary atherosclerosis in this subtype have been reported […].*”

Schmitt et al. reported three similar cases (0.17 %) in a series of 1758 cardiac contrast-enhanced multi-detector CT investigations [24]. The LCA originated together with the RCA from the right sinus of Valsalva, and took a pre-pulmonary course. According to their classification, this would be Type 2a. “*Five anomalies of the LCA main stem were diagnosed, in three of which the LCA main stem originated from the proximal RCA segment, and initial vessel segments travelled in front of the pulmonary trunk with long courses up to 5.5 cm […]. One of these patients presented signs of myocardial ischemia.*”

### Embryology

The organogenesis of the coronary arteries requires a precisely organized array of morphogenetic and molecular events. A comprehensive overview is given for instance in Loukas et al. [13].

Recent studies could demonstrate that the organogenesis of the coronary arteries is based on three fundamental steps: vasculogenesis, angiogenesis and arteriogenesis—subsequently, attachment to the embryonic circulation happens secondarily [4, 25]. Investigations in quail quicken chimera and cell lineage tracing have proposed the proepicardial epithelium (PEO) as a source of the coronary arteries. The PEO is situated between the sinus venosus and the liver and has a mesothelial origin. After their formation, the proepicardial cells migrate to the developing heart, colonize the latter, and induce the formation of the later epicardium. A small population of the PEO is separated from the primordial epicardium and becomes epicardium-derived cells (EPDCs). These EPDCs migrate in an anterior–posterior way, fuse and give rise to a primitive vascular plexus adjacent to the atrioventricular and interventricular grooves as particular preferential areas [9].

These newly organized peritruncal vessels elongate, branch out and invade the aorta [3, 15]. These orifices open mostly into the left and the right sinus, rarely into the posterior sinus [2]. However, the existence of multiple aortal orifices for the coronary arteries has been described which most of them disorganize with further development so that only the definitive coronary arteries persist [8, 21, 28, 29]. It has been noted an association with the coronary development and parasympathetic ganglia, which seem to have a stabilizing function for coronary arteries and promote their maturation [29].

## Conclusion

The first part of the left coronary artery of this case, between its origin and its bifurcation into the LAD and LCx, resembles an intercoronary anastomosis of both the right and left conus branches. This would be the anterior part of a so-called arterial “Circle of Vieussens” [12, 13, 22] or periinfundibular route [17]. Such anastomoses are congenitally determined channels, which are prominent during fetal life and persist until about the eighth postnatal month, when they usually diminish in size [27]. A single coronary artery and an origin of both coronary arteries from the same aortic sinus have been traditionally regarded as having little clinical significance and as compatible with a long and active life [27]; especially, a preinfundibular course as described here is benign in terms of the influence on arterial flow [19]. Variant or anomalous origins and/or courses of the coronary arteries are very often found in major cardiac syndromes such as the transposition of the great arteries or a complete tetralogy of Fallot. Therefore, the formation of a common (mixed) trunk of all elementary coronary trunks can be a challenging finding in pediatric cardiac surgery. Nevertheless, our case presented a variant, preinfundibular course of two of the three elementary coronary trunks (LAD, LCx), without associated alterations of the major cardiac outflows or intrinsic modifications.

However, recognition and angiographic demonstration of such a variation assume the highest priority in a patient undergoing evaluation for direct coronary artery surgery or prosthetic valve replacement [27]. As the course of the LCA in this case shows several quite sharp angulations, these might prevent or at least impede appropriate catheterization either for angiography or (percutaneous) coronary intervention. Zaacks et al. [31] defined a segment angulation of 45° to 89° as moderate and if >90° as extremely angulated. Overall, although benign, such a case with a preinfundibular course of the LCA should be recognized before or during surgery [17].
